# First Evidence that *Ecklonia cava*-Derived Dieckol Attenuates MCF-7 Human Breast Carcinoma Cell Migration

**DOI:** 10.3390/md13041785

**Published:** 2015-03-30

**Authors:** Eun-Kyung Kim, Yujiao Tang, Yon-Suk Kim, Jin-Woo Hwang, Eun-Ju Choi, Ji-Hyeok Lee, Seung-Hong Lee, You-Jin Jeon, Pyo-Jam Park

**Affiliations:** 1 Division of Food Bio Science, College of Biomedical and Health Sciences, Konkuk University, Chungju 380-701, Korea; E-Mails: eunkyungkim@kku.ac.kr (E.-K.K.); yuanxi00@kku.ac.kr (Y.T.); leesh80@kku.ac.kr (S.-H.L.); 2 Korea Nokyong Research Center, Konkuk University, Chungju 380-701, Korea; E-Mails: kimyonsuk@kku.ac.kr (Y.-S.K.); croucard@kku.ac.kr (J.-W.H.); 3 Department of Biotechnology, Konkuk University, Chungju 380-701, Korea; 4 Division of Sport Science, Konkuk University, Chungju, 380-701, Korea; E-Mail: ooj7990@kku.ac.kr; 5 Department of Marine Life Science, Jeju National University, Jeju 690-756, Korea; E-Mails: lee198186@hanmail.net (J.-H.L.); youjinj@cheju.ac.kr (Y.-J.J.)

**Keywords:** *Ecklonia cava*, phlorotannins, dieckol, breast cancer, migration

## Abstract

We investigated the effect of *Ecklonia cava (E. cava)*-derived dieckol on movement behavior and the expression of migration-related genes in MCF-7 human breast cancer cell. Phlorotannins (e.g., dieckol, 6,6′-biecko, and 2,7″-phloroglucinol-6,6′-bieckol) were purified from *E. cava* by using centrifugal partition chromatography. Among the phlorotannins, we found that dieckol inhibited breast cancer cell the most and was selected for further study. Radius™-well was used to assess cell migration, and dieckol (1–100 µM) was found to suppress breast cancer cell movement. Metastasis-related gene expressions were evaluated by RT-PCR and Western blot analysis. In addition, dieckol inhibited the expression of migration-related genes such as matrix metalloproteinase (MMP)-9 and vascular endothelial growth factor (VEGF). On the other hand, it stimulated the expression of tissue inhibitor of metalloproteinase (TIMP)-1 and TIMP-2. These results suggest that dieckol exerts anti-breast cancer activity via the regulation of the expressions of metastasis-related genes, and this is the first report on the anti-breast cancer effect of dieckol.

## 1. Introduction

**Figure 1:**
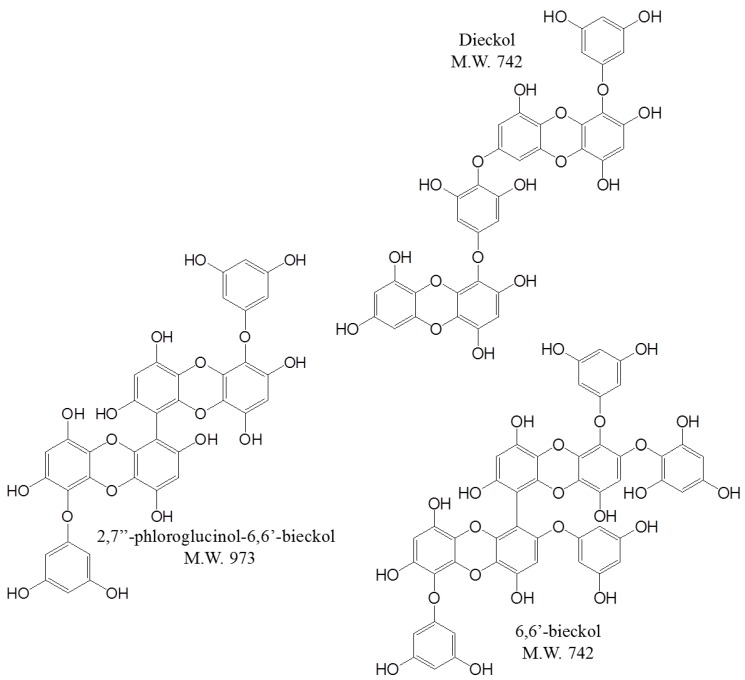
Chemical structures of phlorotannins from *E. cava*.

Brown algae, *E. cava* have been reported to possess various pharmaceutical and biological properties, including anti-cancer, anti-oxidant, anti-allergic, and anti-neurodegenerative effects [1,2,3,4]. Phlorotannins (e.g., dieckol, 6,6′-bieckol, and 2,7″-phloroglucinol-6,6′-bieckol) are the main bioactive components of *E. cava* [5]. Their chemical structures are shown in Figure 1. In particular, dieckol is reported to possess inhibitory activity against ovarian and hepatocellular cancers [6,7]. However, the anti-breast cancer effect of dieckol has not been investigated.

Breast cancer mortality rates have gradually decreased over the last two decades due to newly developed treatment strategies [8]; however, the disease incidence in the United States is still the highest among all cancers in women [9]. It is also the most frequent cause of cancer-related deaths in women [10]. Moreover, breast cancer incidence is rising more rapidly in Asia than in high-risk countries [11]. Therefore, more effective strategies for breast cancer prevention are needed.

Tumor metastasis is a multi-process phenomenon involving the participation of various metastasis-related genes, and almost all cancer deaths are caused by metastasis [12]. Metastasis is the process by which a cancer cell leaves its original location or organ, moves to another site that is not directly connected to the primary site via the circulatory system, and produces a secondary cancer [13]. Matrix metallopeptidases (MMPs) promote tumor progression and metastasis in invasive cancers by cleaving the extracellular matrix (ECM) surrounding the tumor tissue. The degradation of ECM not only assists the migration of metastatic cancerous cells, but also allows for enhanced tumor growth by providing the necessary space [14]. Therefore, MMPs are key targets for many oncogenes [15]. Further, it is noteworthy that the ratio of active to latent forms of MMP-9 increases with tumor progression in invasive cancers. MMP-9 and its family members also promote angiogenesis, a critical process required for tumor cell survival, by degrading the vascular basement membrane interstitium and by releasing sequestered vascular endothelial growth factor (VEGF), a well know angiogenic molecule [16,17]. MMP activity is regulated by endogenous inhibitors, the tissue inhibitors of MMP (TIMPs), which closely bind to MMPs with 1:1 stoichiometry, and MMP activity is correlated with the physiological and pathological processes that coordinate the degradation and accumulation of the ECM [18]. Therefore, blocking the progress of these processes is also a valuable approach to cancer therapy.

Therefore, in the present study, we investigated the effects of dieckol obtained from *E. cava* on the migration behavior and the expression of metastasis-related genes *in vitro* using MCF-7 human breast cancer cell.

## 2. Results and Discussion

### 2.1. Selection of One of the Biological Components in E. cava

**Figure 2:**
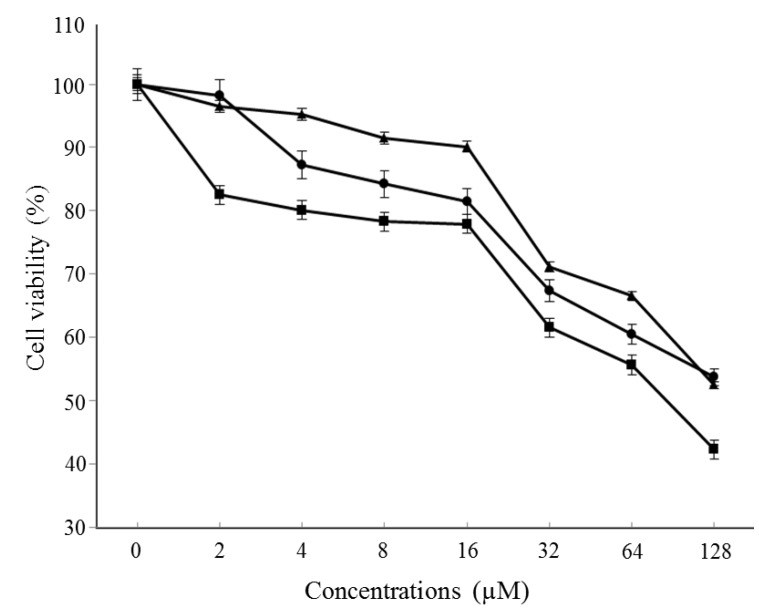
Effect of dieckol on the viability of MCF-7 cells. Cells were treated with various concentrations of dieckol for 48 h followed by MTT assay, and then the absorbance was measured. Cell viability was calculated as the relative absorbance compared to DMSO vehicle control absorbance. 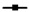, dieckol; 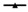, 6,6′-bieckol; 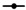, 2,7″-phloroglucinol-6,6-bieckol.

Dieckol showed the greatest inhibitory activity at 2–128 µM, with inhibition percentages ranges of 17.50%–57.69% (Figure 2). Generally, dieckol has been shown to possess inhibitory activity against cancers as well as some enzymes including α-glucosidase and α-amylase *in vitro*, and to alleviate postprandial hyperglycemia in streptozotocin-induced diabetic mice [6,7,19]. In addition, 6,6′-bieckol and 2,7″-phloroglucinol-6,6-bieckol from *E. cava* have been reported to exhibit antioxidant properties [20,21]. However, little information exists concerning which component is effective in breast cancer prevention or therapy. We conducted a cell cytotoxicity assay to determine which molecule most effectively inhibits breast cancer cell viability. MCF-7 cells were treated with three components at the indicated concentrations for 48 h. As a result, dieckol was selected for the anti-breast cancer study.

### 2.2. The Effect of Dieckol on Migration in Human Breast Cancer Cells

**Figure 3:**
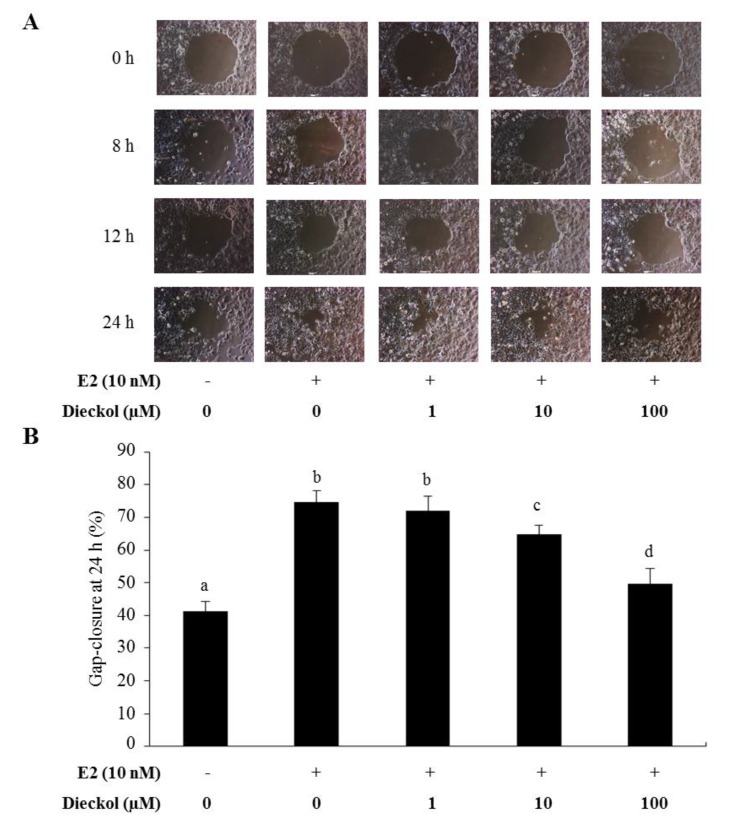
Migration rates of dieckol-treated MCF-7 cells. (**A**) Cell migration time course. Images were captured at the same size after the indicated times (0, 8, 12, and 24 h); (**B**) The gap closure was determined after 24 h using CellProfiler™ software. Values not sharing a common letter are significantly different at *p* < 0.05 by Dunnett’s multiple range test.

To examine the effect of dieckol on breast cancer cell migration, we carried out gap-closure assay using Radius™ wells. To stimulate cell migration, estradiol (E2) was treated on the cell before dieckol treatment. To compare the differences in migratory gap, images were captured at the same size, and the gap-closure was determined after the indicated times (0, 8, 12, and 24 h) and compared between each group. After 24 h, the gap closed by approximately 74.7% in the E2 alone-treated group. As shown in Figure 3, dieckol resulted in significantly lower cell motility in a dose-dependent fashion, compared to that in the E2 alone-treated group. Most cancer deaths are caused by the dissemination of tumors from their primary site. Cancer cells migrate in a highly orchestrated manner that depends on both internal and external signals [22], such as integrins [23], adhesion receptors [24], and chemical signals sensed by chemokine and growth factor receptors [25]. The gap-closure zone assay was recently created to assess cell migration. In this assay, cells are grown on a well bottom around something (e.g., a stopper placed in the middle of the well) that prevents them from growing in one particular region [26]. The experiment begins when the stopper is removed, and the migration of the cells to fill the void in the monolayer is studied. The advantage of the gap-closure assay is that the cell movements can be studied continuously in real time without possible complications of wound-related factors. Due to this advantage, time-series pictures were captured (Figure 3A).

### 2.3. The Effect of Dieckol on the Expression of Migration-Related Genes in Human Breast Cancer Cell

**Figure 4:**
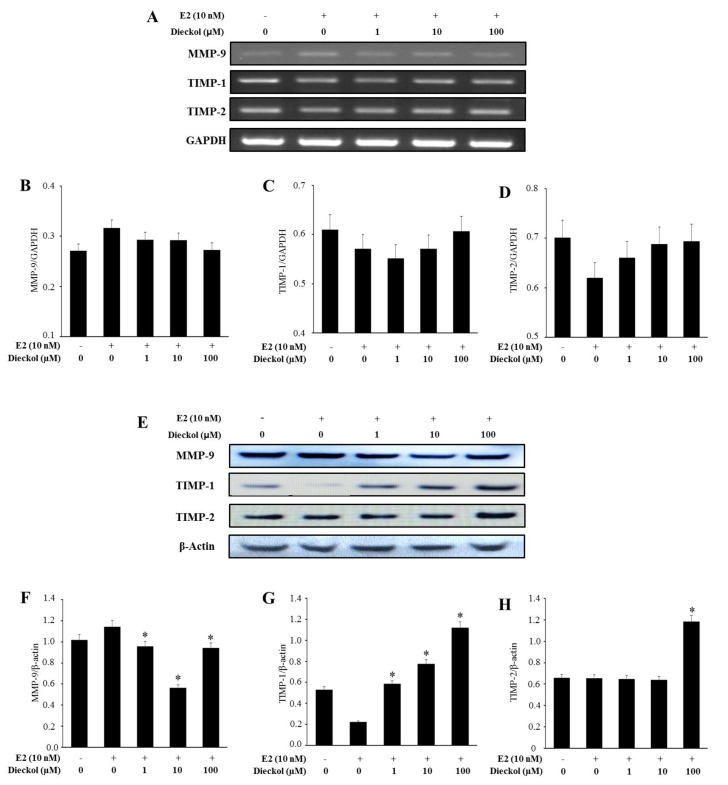
The expression of migration-related genes, based on RT-PCR and Western blot assays. mRNA (**A**–**D**) and protein (**E**–**H**) expression of migration-related genes, quantified by Multi Gauge (Fujifilm, Japan). Cells were treated with various concentrations of dieckol for 48 h with or without E2. The mRNA and protein levels from whole cell lysates were analyzed by RT-PCR or Western blot, respectively. GAPDH and β-actin were used as loading controls. The results were similar in three independent experiments. * Significantly different from the E2 alone group at *p* < 0.05.

As shown in Figure 4A–D, dieckol didn’t significantly affect the mRNA expression. On the contrary, dieckol significantly affected the protein expression (Figure 4E–H). Briefly, the protein expression of MMP-9 was significantly inhibited (Figure 4E,F). On the other hand, the protein expressions of TIMP-1 and TIMP-2 were significantly increased in dieckol-treated human breast cancer cell (Figure 4E,G,H). Meanwhile, the protein expression of MMP-9 was in a dose-independent manner, while TIMPs were in a dose-dependent manner. The effect of the concentrations on the genes should be further investigated. Cancers exhibit two modes of motility: adhesion receptor-mediated basal movement and a faster motility in response to soluble growth factors [27]. Growth factor receptor-mediated movement is an essential driver of cancer cell dissemination via metastasis [28,29]. Thus, understanding the key molecular controls of this behavior should provide novel targets to limit initial or secondary dissemination. From this result, it was clearly seen that dieckol stimulated the protein expression of TIMPs. TIMPs are glycoproteins that are natural inhibitors of MMPs as well as a group of peptidases, and are involved in ECM degradation. Therefore, these data suggest that dieckol suppresses cell migration by up-regulating TIMP-1 and TIMP-2 levels in MCF-7 human breast cancer cell.

### 2.4. The Effect of Dieckol on the Expression of the Angiogenesis-Related Gene VEGF in Human Breast Cancer Cell

**Figure 5:**
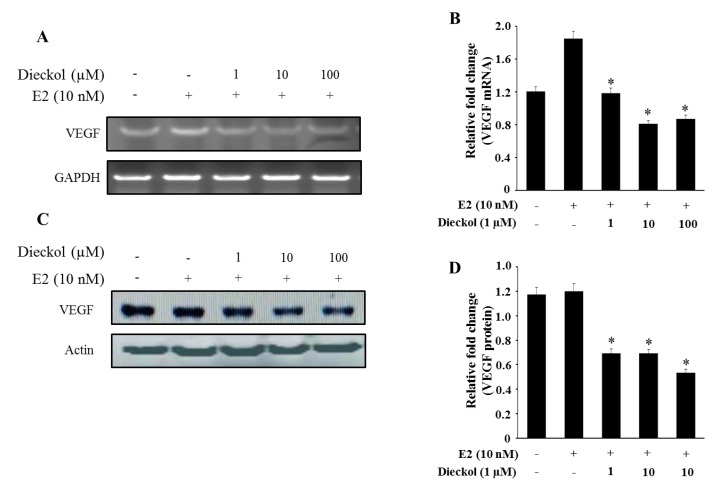
The mRNA and protein expression of VEGF. mRNA (**A**,**B**) and protein (**C**,**D**) expression of VEGF were quantified by Multi Gauge (Fujifilm, Japan). Cells were treated with various concentrations of dieckol for 48 h with or without E2. The mRNA and protein levels from whole cell lysates were analyzed by RT-PCR or Western blot, respectively. GAPDH and β-actin were used as a loading control. The results were similar in three independent experiments. * Significantly different from the E2 alone group at *p* < 0.05.

Dieckol significantly inhibited the mRNA (Figure 5A,B) and protein (Figure 5C,D) expression of VEGF in a dose-dependent manner. The angiogenic response is induced by growth factors such as VEGF, basic fibroblast growth factor (bFGF), platelet-derived growth factor (PDGF), and chemokines [30]. ECM is degraded by several matrix-degrading enzymes [31], such as MMPs, which are produced by stromal cells, endothelial cells, or tumor cells themselves [32]. Therefore, VEGF has been recognized as a key potential target for pharmacological inhibition of tumor angiogenesis, and from Figure 5, we confirmed that dieckol possessed the inhibitory effect of VEGF.

Recently, Kim *et al*., reported the effect of phloroglucinol which is also a phlorotannin component on breast cancer cells. Phloroglucinol effectively inhibited EMT (epithelial-mesenchymal cell transition)-related genes such as, FN1, VIM, *N*-cadherin, SNAIL, *etc*. EMT is also related to cancer metastasis, therefore, the investigation has given us motivation to do further study to investigate the precise cancer inhibitory mechanism of dieckol. All in all, it was proven that brown algae-derived phlorotannins, including dieckol and phloroglucinol, possessed an inhibitory effect on cancer metastasis. However the investigation of the precise mechanism of each component is needed [33].

## 3. Experimental Section

### 3.1. Materials

Marine edible brown seaweed, *E. cava*, was collected from Jeju Island off the coast of the Republic of Korea. Fresh *E. cava* was washed three times with water to remove salt, sand, and epiphytes. The cleaned *E. cava* was ground and sifted through a 50 mesh standard testing sieve after drying in a freeze dryer SFDSMO6 (Samwon Freezing Engineering Co., Gyeonggi, Korea). The dried *E. cava* was stored in a refrigerator until use. All solvents used for the preparation of crude samples were of analytical grade (Daejung Chemicals & Metals Co., Seoul, South Korea). HPLC grade solvents were purchased from Burdick & Jackson (MI, USA). MCF-7 human breast cancer cell line was obtained from the Korean Cell Line Bank (Seoul, Korea; KCLB numbers: 30022). RPMI 1640 media for the cells and TRIzol reagent for RNA extraction were from Invitrogen (Carlsbad, CA, USA). E2 was purchased from Sigma (St. Louis, MO, USA). Primary antibodies including MMP-9 (SC-10737), TIMP-1 (Cat. SC-6823), TIMP-2 (Cat. SC-6835), VEGF (Cat. SC-152), and β-actin (SC-1616) were purchased from Santa Cruz Biotechnology Inc. (Santa Cruz, CA, USA) as well as the peroxidase-conjugated secondary antibodies including anti-gout (SC-2020), and anti-mouse (SC-2005).

### 3.2. Extraction and Isolation of the Major Components of E. cava

The phlorotannins were isolated according to the previously reported method [34,35]. The dried *E. cava* powder (500 g) was extracted using 5 L of 80% aqueous methanol three times at room temperature. The liquid layer was obtained via filtration, and the filtrate was concentrated using an evaporator under reduced pressure. The extract was suspended in H_2_O, and the aqueous layer was partitioned with ethyl acetate (EtOAc). The EtOAc extract was mixed with celite. The mixed celite was then dried and packed into a glass column, and subsequently eluted in the following order: hexane, dichloromethane, diethyl ether, and butanol. The diethyl ether fraction was subjected to Sephadex LH-20 column chromatography using a stepwise gradient with chloroform/methanol (2/1 to 1/1 to 0/1) solvents system. The dieckol, 6,6′-bieckol, and 2,7″-phloroglucinol-6,6′-bieckol were purified by HPLC using a Waters HPLC system equipped with a Waters 996 photodiode array detector and C_18_ column (J’sphere ODS-H80, 150 × 20 mm, 4 μm; YMC Co., Kyoto, Japan) by stepwise elution with a methanol-water gradient. Finally, the purified compounds were identified by comparing the ^1^H and ^13^C-NMR spectral data with those in the existing literature [21,34,35]. The chemical structures of the three phlorotannins are indicated in Figure 1.

### 3.3. Cell Culture

The MCF-7 human breast cancer cell line was cultured in RPMI 1640 media supplemented with 10% FBS and 1% penicillin/streptomycin in a 5% CO_2_ atmosphere at 37 °C. The cells were seeded at a density of 5.0 × 10^5^ in a 6-well culture plate. After 24 h, the cells were treated with or without 10 nM of E2 or 1–100 µM of dieckol extracts in media for 48 h and then harvested.

### 3.4. Cytotoxicity Assay

Cell viability was determined by 3-(4,5-dimethylthiazol-2yl)-2,5-diphenyltetrazolium bromide (MTT) assay in 96-well plates, as previously described [36]. Cells were incubated with various concentrations of dieckol for 48 h followed by MTT for 4 h, and then 100 μL isopropanol (in 0.04 *N*-hydrochloric acid) was added to dissolve the formazan crystals. The absorbance was read at 570 nm using a spectrophotometer (Tecan, Switzerland). Cell viability was calculated as the relative absorbance compared to DMSO vehicle control absorbance [36].

### 3.5. Gap Closure Migration Assay

We performed the migration assay using the Radius™ 24-well from Cell Biolabs, Inc (San Diego, CA, USA). For the analysis, 500 µL Radius™ gel pretreatment solution was slowly added to each well by carefully pipetting down the wall of the well, and then the plate was covered and incubated at room temperature for 20 min. Radius™ gel pretreatment solution was carefully aspirated from the wells, and 500 µL Radius™ wash solution was added to each well. The cells were harvested and resuspended in culture medium at 0.2 × 10^6^ cells/mL. Radius™ wash solution was carefully aspirated from the wells, and 500 µL of the cell suspension was added to each well by carefully pipetting down the wall of the well. The plate was transferred to a cell culture incubator for 24 h to allow firm attachment. After 24 h, media was aspirated from each well and washed 3 times with 0.5 mL fresh media. A sufficient amount of 1× Radius™ gel removal solution for all wells was prepared by diluting the stock 1:100 in culture media. The media was aspirated from the wells, and 0.5 mL 1× Radius™ gel removal solution was added to each well and washed 3 times with 0.5 mL fresh media. After the final washing was complete, 1 mL of complete medium with dieckol (1–100 µM) was added to each well, and a photo was taken at 0, 8, 12, and 24 h. To compare differences in the migratory gap, images were captured at the same size, and gap closure was determined after the indicated times (0, 8, 12, and 24 h) using CellProfiler™ software (Broad Institute, Cambridge, MA, USA).

### 3.6. RNA Isolation and mRNA Expression Analysis

For RT-PCR, total cellular RNA was isolated from cells using TRIzol, according to the manufacturer’s protocol. The first-strand complementary DNA (cDNA) was synthesized using Superscript II reverse transcriptase (Invitrogen, Carlsbad, CA, USA). PCR was performed as previously described with primers for MMP-9 (GenBank accession no. AK301446.1; s 5ʹ-CGA CGT CTT CCA GTA CCG AG-3ʹ; as 5ʹ-GTT GGT CCC AGT GGG GAT TT-3ʹ), TIMP-1 (GenBank accession no. AK311937.1; s 5ʹ-CAA GAT GAC CAA GAT GTA TAA AGG-3ʹ; as 5ʹ-AAC AGT GTA GGT CTT GGT GAA G-3ʹ), TIMP-2 (GenBank accession no. AL110197.1; s 5ʹ-CAG CTT TGC TTT ATC CGG GC-3ʹ; as 5ʹ-ATG CTT AGC TGG CGT CAC AT-3ʹ), and VEGF (GenBank accession no. AB209485.1; s 5ʹ-AGG GCA GAA TCA TCA CGA AG-3ʹ; as 5ʹ-TTT CTC CGC TCTGAG CAA GG-3ʹ). GAPDH (GenBank accession no. AB062273.1; s 5ʹ-CCA TGG GGA AGG TGA AGG TC-3ʹ; as 5ʹ-AAA TGA GCC CCA GCC TTC TC-3ʹ) was used as an internal control. The conditions for RT-PCR were similar to ones that have been previously described [37].

### 3.7. Western Blot Analysis

Cell extracts were prepared by the detergent lysis procedure, as described elsewhere [38]. Samples of protein (40 μg) were used for electrophoresis using Novex 4%–12% Bis-Tris gels (Life Technologies, Carlsbad, CA, USA) and then transferred to nitrocellulose membranes for 7 min in the iBlot dry blotting system (Life Technologies, Carlsbad, CA, USA). The transferred proteins were blocked overnight at 4 °C with clear milk (Thermo Scientific, IL, USA). Blots were subsequently incubated with primary antibodies diluted 1:2000 in 1× TBST for 1 h. Goat anti-rabbit or goat anti-mouse horseradish peroxidase conjugated secondary antibodies (Santa Cruz Biotechnology, Dallas, TX, USA) were used at 1:2000 dilution in 1× TBST. Blots were treated with Western Lightning Western Blot Chemilluminescence Reagent (Advansta, CA, USA) and the proteins were detected by autoradiography. Equal protein loading was ascertained by ponceau S staining of blotted membranes as well as Western blotting with β-actin bodies. Immunodetection was done using an enhanced chemiluminescence detection kit (Amersham Pharmacia, Piscataway, NJ, USA).

### 3.8. Data Analysis

The results from the experiments shown are summaries of the data sourced from at least three experiments. All of the data are presented as the mean ± SE. Statistical analyses were performed using SAS statistical software (SAS Institute, Cray, NC, USA). Treatment effects were analyzed using one-way analysis of variance, followed by Dunnett's multiple range test. The results *p* < 0.05 were used to indicate significance.

## 4. Conclusions

In the present study, we demonstrated for the first time the effects of dieckol derived from *E. cava* ethanol extracts on human MCF-7 breast cancer cell *in vitro*. Cell cytotoxicity was determined using an MTT assay, and migration was detected using a gap closer assay. Further, we evaluated the mRNA and protein expression levels of MMP-9, TIMP-1, TIMP-2, and VEGF using RT-PCR and Western blot, respectively. The results of the present study characterize the signaling cascades that mediate the anti-metastatic activity of dieckol in the human MCF-7 breast cancer cell line. Dieckol was found to significantly suppress the migration of MCF-7 cells and inhibit the expression of MMP-9 and VEGF. On the contrary, TIMP-1 and TIMP-2 were increased in MCF-7 cell by dieckol treatment. These findings demonstrate the biological activity of dieckol in an *in vitro* model of cancer metastasis, and show that dieckol may be a promising new therapeutic in breast cancer therapy.
